# Embryonic and larval development in the Midas cichlid fish species flock (*Amphilophus spp.*): a new evo-devo model for the investigation of adaptive novelties and species differences

**DOI:** 10.1186/s12861-015-0061-1

**Published:** 2015-02-26

**Authors:** Claudius F Kratochwil, Maggie M Sefton, Axel Meyer

**Affiliations:** Zoology and Evolutionary Biology, Department of Biology, University of Konstanz, Konstanz, Germany; Zukunftskolleg, University of Konstanz, Konstanz, Germany; International Max Planck Research School for Organismal Biology, University of Konstanz, Konstanz, Germany

**Keywords:** Teleostei, Ontogeny, Parallel evolution, Phenotypic diversification, Cichlidae, Pigmentation, Melanophore, Xanthophore, *Amphilophus citrinellus*, *Amphilophus xiloaensis*

## Abstract

**Background:**

Central American crater lake cichlid fish of the Midas species complex (*Amphilophus spp.*) are a model system for sympatric speciation and fast ecological diversification and specialization. Midas cichlids have been intensively analyzed from an ecological and morphological perspective. Genomic resources such as transcriptomic and genomic data sets, and a high-quality draft genome are available now. Many ecologically relevant species-specific traits and differences such as pigmentation and cranial morphology arise during development. Detailed descriptions of the early development of the Midas cichlid in particular, will help to investigate the ontogeny of species differences and adaptations.

**Results:**

We describe the embryonic and larval development of the crater lake cichlid, *Amphilophus xiloaensis,* until seven days after fertilization*.* Similar to previous studies on teleost development, we describe six periods of embryogenesis - the zygote, cleavage, blastula, gastrula, segmentation, and post-hatching period. Furthermore, we define homologous stages to well-described teleost models such as medaka and zebrafish, as well as other cichlid species such as the Nile tilapia and the South American cichlid *Cichlasoma dimerus*. Key morphological differences between the embryos of Midas cichlids and other teleosts are highlighted and discussed, including the presence of adhesive glands and different early chromatophore patterns, as well as variation in developmental timing.

**Conclusions:**

The developmental staging of the Midas cichlid will aid researchers in the comparative investigation of teleost ontogenies. It will facilitate comparative developmental biological studies of Neotropical and African cichlid fish in particular. In the past, the species flocks of the African Great Lakes have received the most attention from researchers, but some lineages of the 300–400 species of Central American lakes are fascinating model systems for adaptive radiation and rapid phenotypic evolution. The availability of genetic resources, their status as a model system for evolutionary research, and the possibility to perform functional experiments including transgenesis makes the Midas cichlid complex a very attractive model for evolutionary-developmental research.

## Background

**Figure 1:**
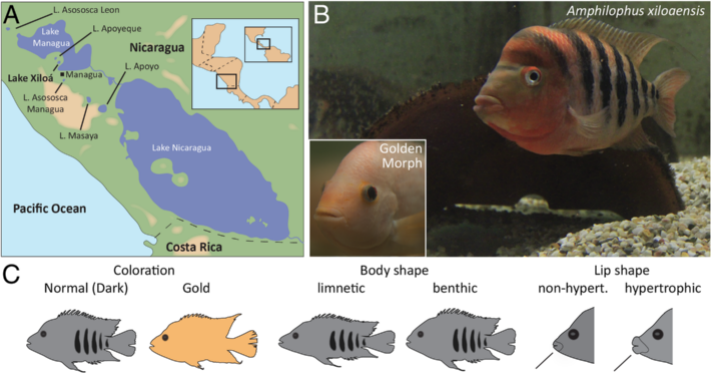
**Range and prominent phenotypic differences of members of the Midas cichlid species complex. (A)** Map of the Pacific coast of Nicaragua in Central America. Besides the large Nicaraguan lakes (Managua and Nicaragua), multiple crater lakes (Asososca Leon, Apoyeque, Xiloá, Asosoca Managua, Masaya and Apoyo) have appeared in the course of the last 25,000 years. These crater lakes have been colonized by Midas cichlids from the large lakes, resulting in new species. **(B)** Midas cichlids from Lake Xiloá, *Amphilophus xiloaensis*, the focal species of this study. **(C)** Three selected traits that are interesting from an evolutionary-developmental angle. In the large lakes and in many crater lakes, cichlid species and morphs show differences in coloration, body shape and lip shape.

Cichlids are famous for their astonishing rate of phenotypic diversification and speciation. With over 2000 described species, cichlid fish form one of the most diverse and species-rich groups of animals [1]. Lacustrine cichlids in Africa and in the Neotropics are well-known examples of adaptive radiations [2-4]. In particular, the cichlid radiations in Nicaraguan crater lakes (Figure 1, Table 1) provide a promising opportunity to study the early stages of speciation and diversification. This is because members of the Midas cichlid species complex (*Amphilophus spp.* or *Amphilophus citrinellus spp.*) have diverged repeatedly in several crater lakes, both sympatrically and allopatrically, often within a few thousand years [2,5-7]. Little is known so far about the molecular and developmental mechanisms that drive the observed phenotypic diversity between recently diverged species. The Midas cichlid complex underwent a rapid diversification within very short time spans (between 2000 and 25,000 years) and, interestingly, repeatedly evolved several adaptive traits (hypertrophied lips, elongated body shapes, dental innovations) in parallel in multiple crater lakes (Figure 1, Table 1). Therefore, Midas cichlids are an excellent model system for the comparative study of the phenotype-genotype relationship.

**Table 1:** **Summary of discovered Midas cichlid species**

**Species**	**Described by**	**Endemic to lake**	**Coloration**	**Body shape**	**Lip shape**	**Genome availability**
*A. citrinellus*	Günther, 1864 [9]	-	normal or gold	benthic	non-hypertrophic^#^ or hypertrophic*	high quality (draft genome in [6])
*A. labiatus*	Günther, 1864 [8]	-	normal or gold	limnetic	hypertrophic	low coverage [6]
*A. zaliosus*	Barlow & Munsey 1976 [10]	Apoyo	normal	limnetic	non-hypertrophic	low coverage [6]
*A. astorquii*	Staufer et al., 2008 [12]	Apoyo	normal	benthic	non-hypertrophic	low coverage [6]
*A. chancho*	Staufer et al., 2008 [12]	Apoyo	normal	benthic	non-hypertrophic	low coverage [6]
*A. flaveolus*	Staufer et al., 2008 [12]	Apoyo	normal	benthic	non-hypertrophic	low coverage [6]
*A. globosus*	Geiger et al., 2010 [11]	Apoyo	normal	benthic	non-hypertrophic	low coverage [6]
*A. supercilius*	Geiger et al., 2010 [11]	Apoyo	normal	benthic	non-hypertrophic	low coverage [6]
*A. amarillo*	Staufer et al., 2002 [13]	Xiloá	normal (gold rare or absent)	benthic	non-hypertrophic	low coverage [6]
*A. sagittae*	Staufer et al., 2002 [13]	Xiloá	normal or gold	limnetic	non-hypertrophic	low coverage [6]
*A. xiloaensis*	Staufer et al., 2002 [13]	Xiloá	normal or gold	benthic	non-hypertrophic	low coverage [6]
*A. viridis*	Recknagel et al. 2013 [14]	Xiloá	normal	benthic	non-hypertrophic	low coverage [6]
*A. tolteca*	Recknagel et al. 2013 [14]	Asososca Managua	normal or gold	benthic or limnetic	non-hypertrophic	-

The 13 species of the Midas cichlid species complex listed with their range, a selection of their observed phenotypic variations (coloration, body and lip shape) and availability of a sequenced genome (^#^Lake Managua and Nicaragua, *crater lakes).

The Midas cichlid species complex currently includes 13 described species (Table 1). Two ancestral “source” species occur in the big lakes, Lake Managua and Lake Nicaragua - *Amphilophus labiatus* [8] and *A. citrinellus* [9]. These two species repeatedly and independently colonized the much younger crater lakes of Nicaragua and gave rise to several endemic species. Since the late 1970s, many endemic crater lake species have been described. Six species, *A. zaliosus*, *A. astorquii*, *A. chancho*, *A. flaveolus*, *A. globosus* and *A. supercilius* were recently described and are endemic to crater Lake Apoyo [10-12]. Four other species of this species complex are endemic to crater Lake Xiloá *(A. amarillo*, *A. sagittae*, *A. xiloaensis and A. viridis)* [13,14] and one to Lake Asososca Managua, *A. tolteca* [14]. Despite these numerous recently-described species, more Midas cichlids certainly await formal species description [15,16].

The focal species of this study, *Amphilophus xiloaensis,* was first described in 2002 [13] and is endemic to Lake Xiloá (Figure 1B). This crater lake is estimated to be approximately 6100 years old [5,17]. Lake Xiloá has the greatest fish species diversity of any of the Nicaraguan crater lakes [18], including four Midas cichlids with an exceptionally high haplotype diversity relative to the lake’s age [19]. Since these species are so young, they share ancient polymorphisms [7] and some hybridization still occurs, as has been reported for African cichlids [20,21].

Many studies have assessed the early ontogeny of fishes in classic model organisms such as zebrafish, *Danio rerio* [22]; medaka, *Oryzias latipes* [23]; stickleback, *Gasterosteus aculeatus* [24] and rainbow trout, *Oncorhynchus mykiss* [25]. However, there have been only a few studies on cichlid fishes so far, most of which deal with the development of African species such as *Oreochromis niloticus, Oreochromis mossambicus*, *Labeotropheus fuelleborni* and *Labeotropheus trewavasae* [26-28]. Developmental studies of Neotropical cichlids have also been pursued, including a very detailed description of the development of the South American cichlid *Cichlasoma dimerus* [29-34]. Because ontogeny can differ strongly among species, there is a need for more developmental work [35].

Midas cichlids are a famous example of parallel evolution and rapid diversification [36-39]. This makes them interesting, not only from an evolutionary and ecological standpoint, but also from a developmental “evo-devo” perspective. A detailed description of the embryonic development of the Midas cichlid is still lacking. The present study aims to be a foundation for future studies examining the genetic and developmental factors that lead to phenotypic diversification among an extremely young species of a particularly species-rich lineage of cichlid fish.

## Results

### Description of the early development of the Midas cichlid

We document in detail the early development of the Midas cichlid, *Amphilophus xiloaensis*, during the first seven days following fertilization at 28°C. We illustrate and discuss the main features of 30 developmental stages in the first seven days of development and compare them to previous descriptions of teleost development. As a reference, we mainly use the well-documented developmental staging of the zebrafish [22], the medaka [23] and two of the most comprehensive descriptions of cichlid development - the Nile tilapia *Oreochromis niloticus* [26] and the South American cichlid *Cichlasoma dimerus* [29]. Lastly, we discuss differences in the rate of early development, which is comparatively slow in Midas cichlids. The age of the embryos is given in hours post fertilization (h) or days after fertilization (d) at 28°C, unless otherwise indicated.

### Zygote period (0–1.75 h)

**Figure 2:**
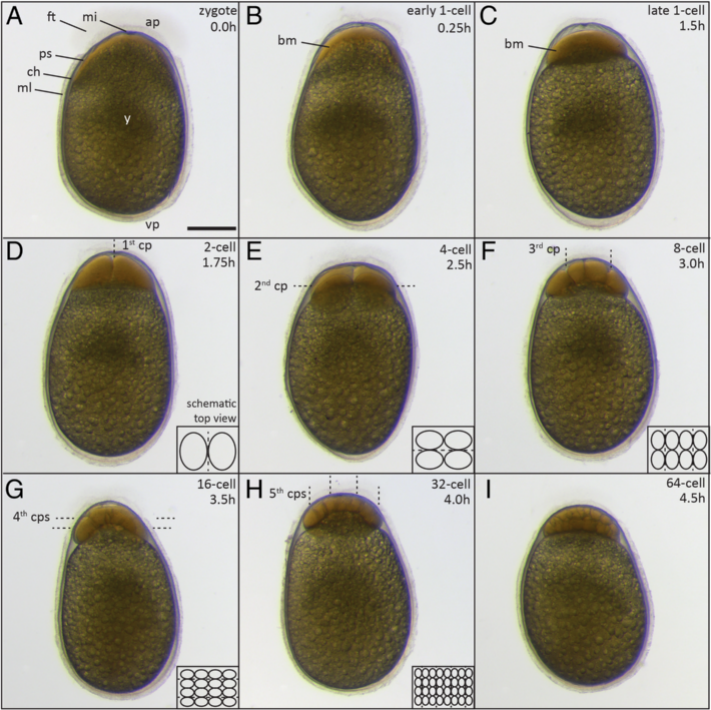
**Embryos during cleavage and blastula stages. (A)** zygote stage (0 h); **(B)** early 1-cell stage (0.25 h); **(C)** late 1-cell stage (1.5 h); **(D)** 2-cell stage (1.75 h); **(E)** 4-cell stage (2.5 h); **(F)** 8-cell stage (3 h); **(G)** 16-cell stage (3.5 h); **(H)** 32-cell stage (4 h); **(I)** 64-cell stage (4.5 h). Schemes illustrate the position of cells and cleavage planes from a top-down view **(D-H)**. Abbreviations: ap, animal pole; bm, blastomeres; ch, chorion; cp(s), cleavage plane(s); ft, filament tuft; mi, micropyle; ml, mucous layer; ps, perivitelline space; vp, vegetal pole; y, yolk. Scale bar = 500 μm.

**Figure 3:**
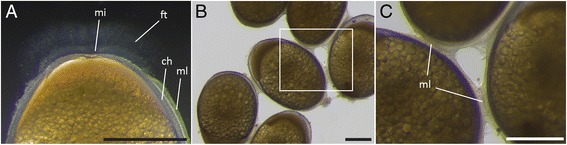
**Micropylar region and mucous layer. (A)** At the one-cell stage, the micropylar region is surrounded by the filament tuft. **(B, C)** The mucous layer adheres the eggs to the substrate and/or to one another at low **(B)** and high magnification **(C)**. Abbreviations: ch, chorion; mi, micropyle; ft, filament tuft; ml, mucous layer. Scale bar = 500 μm.

Unfertilized or newly-fertilized eggs of *A. xiloaensis* have an ovoid shape, with the longitudinal axis longer (2.14 ± 0.09 mm) than the transverse axis (1.42 ± 0.07 mm) and the animal pole narrower than the vegetal pole (Figure 2A). The egg is surrounded by the chorion, a translucent envelope that sticks closely to the egg (Figures 2A, 3A). This persists throughout later developmental stages, when there is almost no perivitelline space between the chorion and the vitellus (egg yolk). The vitellus is composed of large dark-yellow yolk globules/platelets of varying sizes (0.01-0.09 mm), giving it a grainy appearance, as reported previously for the Midas cichlid and closely-related Neotropical cichlids [35,40,41] (Figures 2A, 3A). The micropyle, the pore in the membrane that guides sperm to the oocyte [42], has a funnel or cone-shaped configuration. It is surrounded by a tuft of filament that can best be observed with dark field illumination (Figure 3A), and can only be seen until the first four to six cell divisions (Figure 2A-I). After spawning (both natural and by stripping) the eggs stick to each other and to the substrate, or to the petri dish under laboratory conditions, by a mucous secretion (Figure 3B, C). In contrast to zebrafish [22], the chorion does not swell and lift away from the fertilized egg during the zygote period, which lasts until the first cleavage occurs around 1.75 h (28°C).

*One-cell stage (0 h)*. Fertilization induces cytoplasmic movements at the animal pole, where the blastodisc increases in volume and replaces the yolk (Figure 2B, C). The blastodisc gradually segregates from the yolk and forms a more prominent, clearly defined cell at 1.5 hours, and the perivitelline space becomes visible. The cytoplasm is uniform, but darker than in other teleosts [22,29] (Figure 2B, C).

### Cleavage period (1.75-5 h)

After 1.75 hours, cleavages occur every 35 minutes (at 28°C). The cleavage mode is meroblastic (incomplete) discoidal, as in other teleosts. The six synchronously-occurring divisions of this period result in stereotyped arrays of blastomeres, as reported previously [22,23,29] (Figure 2D-I). The egg is telolecithal and the meroblastic divisions keep a connection between yolk and blastodisc during the cleavage period.

*Two-cell stage (1.75 h)*. The first cleavage furrow is vertically oriented (meridional), dividing the blastodisc into two cells (blastomeres) of equal size. Both cells stay connected to the underlying yolk (meroblastic cleavage) (Figure 2D).

*Four-cell stage (2.5 h)*. In the second division, the cleavage plane is oriented in a right angle to the first cleavage plane, resulting in four blastomeres arranged in a 2 × 2 array if viewed from the animal pole (Figure 2E).

*Eight-cell stage (3 h)*. The third set of cleavages occurs in two planes parallel to the first cleavage plane, dividing the four blastomeres into eight blastomeres. They are arranged in a 2 × 4 array. Viewed laterally, only four cells are visible (Figure 2F).

*16-cell stage (3.5 h)*. The fourth cleavage plane also occurs on two planes, this time parallel to the second cleavage plane. The two rows of four blastomeres are divided into four rows of four blastomeres (4 × 4 array) (Figure 2G).

*32-cell stage (4 h)*. The fifth set of cleavages generates a 4 × 8 array of cells, although the pattern is less stereotypic than in previous stages. All cells are still in contact with the yolk. Often, the blastodisc curves around the yolk, shaping the underlying yolk in a dome-like structure (Figure 2H).

*64-cell stage (4.5 h)*. During the sixth set of divisions, cells start to be cleaved completely from the others, forming a second layer of cells on top of those that are still connected to the yolk (marginal cells). Unlike in previous stages, there are no regularly-patterned cleavage planes or stereotypical cell arrangements (Figure 2I).

### Blastula period (5-20 h)

**Figure 4:**
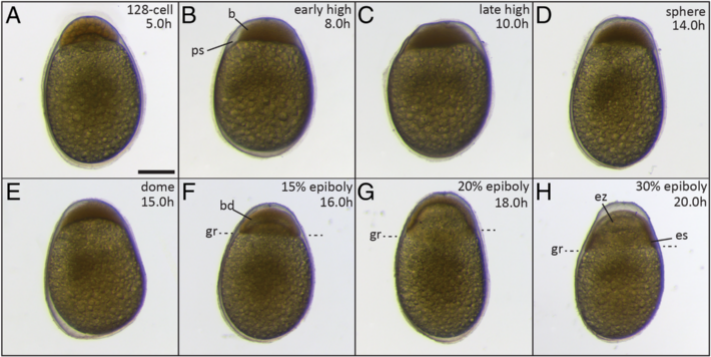
**Embryos during late blastula and early gastrulation phases. (A)** 128-cell stage (5 h); **(B)** early high stage (8 h); **(C)** late high stage (10 h); **(D)** sphere stage (14 h); **(E)** dome stage (15 h); **(F)** 15% epiboly (16 h); **(G)** 20% epiboly (18 h). **(H)** 30% epiboly (20 h). The position of the germ ring (gr in F-H) is indicated by the dashed lines. Abbreviations: b, blastodisc; bd, blastoderm; es, embryonic shield, ez, evacuation zone, gr, germ ring; ps, perivitelline space. Scale bar = 500 μm.

The blastula period extends from the 128-cell stage until gastrulation. Cleavages occur with increasing irregularity. The blastodisc acquires a more uniform appearance, and starts to thin and spread around the yolk (epiboly). At 50% epiboly, when half of the yolk is covered by the blastodisc, gastrulation begins (Figure 4).

*Morula stage (5 h)*. Cleavages continue to occur. As in the 64-cell stage divisions, no clear cleavage planes can be identified. The seventh, eighth and ninth cleavages result in 128, 256 and 512 blastomeres, respectively. Consequently, cells gradually become smaller, without a clear increase in the size of the blastodisc (Figure 4A).

*High stage (8/10 h)*. The blastodisc is a thick, ball-shaped structure on top of the yolk, the hallmark of this stage compared to later stages (Figure 4B-C).

*Sphere stage (14 h)*. After the high stage, the blastodisc gradually flattens, resulting in a spherical shape (Figure 4D).

*Dome stage (15 h)*. The flattening of the blastodisc continues, starting to cover the top of the yolk, which bulges towards the animal in a dome-like shape, as described for zebrafish by Kimmel *et al*. [22] (Figure 4E).

*Early epiboly stages (15% - 16 h/20% - 18 h)*. The blastodisc, which gradually transforms into a uniformly thick layer, starts to cover the yolk and is now called the blastoderm. This stage can be measured by the percentage of epiboly. We defined two stages of early epiboly depending on how far the blastoderm margin (the germ ring) has spread over the yolk: 15% and 20%, measured by the ratio between the distances between the animal pole and blastoderm margin, and between the animal and vegetal pole (Figure 4F-G).

### Gastrula period (20-34 h)

**Figure 5:**
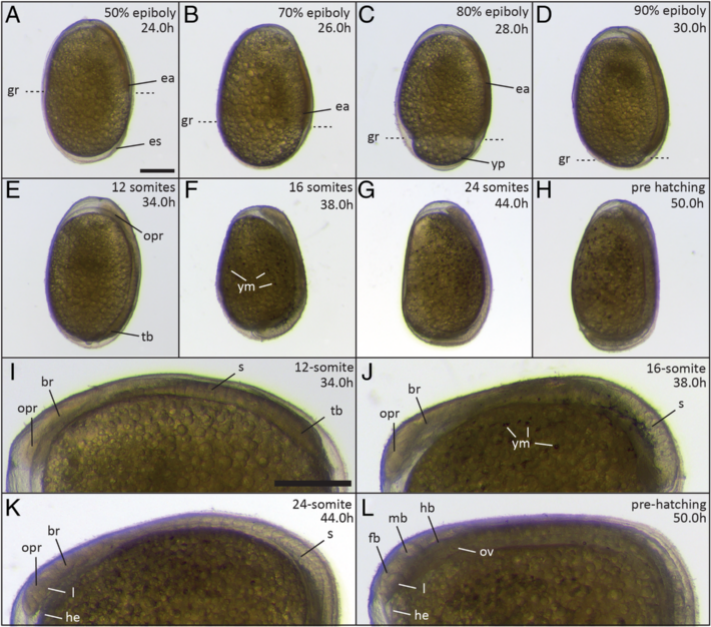
**Embryos during gastrulation and segmentation stages. (A)** 50% epiboly (24 h); **(B)** 70% epiboly (26 h); **(C)** 80% epiboly (28 h); **(D)** 90% epiboly (30 h); **(E, I)** 8 somites (34 h); **(F, J)** 16 somites (38 h); **(G, K)** 24 somites (44 h); **(H, L)** Pre-hatching stage (50 h). The position of the germ ring (gr in A-D) is indicated by the dashed lines. Abbreviations: br, brain; ea, embryonic axis; fb, forebrain; gr, germ ring; he, heart; hb, hindbrain; l, lens; ym, yolk melanophores; mb, midbrain; opr, optic primordium; ov, otic vesicle; s, somites; tb, tailbud; yp, yolk plug; Scale bar = 500 μm.

When 30% epiboly is reached, cells start to accumulate at one position on the dorsal side of the blastoderm margin. Gastrulation starts at this position by the involution of cells, eventually giving rise to the three germ layers. Epiboly continues until the blastoderm completely covers the yolk. In contrast to zebrafish segmentation, the next period of development, starts before 100% epiboly is reached (Figures 4H, 5A-C).

*30% epiboly – shield stage (20 h)*. When epiboly has progressed to 30% of the yolk, a thickening appears at one position of the blastoderm margin (now defined as the dorsal side). This thickening is referred to as the “shield” [22] and is the result of cellular movements. Gastrulation and cell involution take place in this part of the blastoderm (Figure 4H). In zebrafish, this stage occurs later, at 50% epiboly. Due to epiboly and convergence towards the embryonic shield, the blastoderm becomes thin at the animal pole. This is referred to as the evacuation zone, because of the reduced number of cells [22] (Figure 4H).

*Late epiboly stages (50% - 24 h/70% - 26 h/80% - 28 h)*. At 50% epiboly, the dorsal side of the blastoderm thickens further and the future embryonic axis becomes visible, with the anterior end in the direction of the animal pole (Figure 5A-C). After 70% of the yolk is covered, the speed of epiboly continues at a constant rate of about 5% per hour (three times slower than in zebrafish [22]). Later stages of epiboly are characterized by the presence of the yolk plug, the section of yolk at the vegetal pole that has not yet been encompassed by the blastoderm (Figure 5C).

### Segmentation period (30-66 h)

During the segmentation period, the long axis of the embryo forms and extends further, even before epiboly is complete. Structures including the somites, tail, eye and auditory vesicle begin to take shape. Additionally, the brain starts to grow in size. Pigmentation appears first on the yolk sac and later on the body axis. (Figure 5D-H).

*6-somite stage/90% epiboly (30 h)*. Somitogenesis starts before the end of epiboly. At 90% epiboly, eight somites have formed and the tail bud appears at the posterior end of the body axis. At the anterior end of the embryo, the brain primordium, without visible morphological subdivisions, and the optic primordia, which evaginates from the future diencephalon part of the brain primordium are visible. The otic (acoustic) vesicle is forming in the posterior head region. (Figure 5D).

*12-somite stage (34 h)*. At the 12-somite stage, epiboly is finished and the entire yolk is covered by blastoderm. The tail bud and optic primordia become more prominent (Figure 5E, I).

*16-somite stage (38 h)*. At the 16-somite stage, the first melanophores appear on top of the yolk sac as well as in the posterior part of the embryo. The tail extends further and starts to curl inside the chorion. The pericardial sac forms between the anteriormost region of the yolk and the head region, slightly lifting the head from the yolk (Figure 5F, J).

*24-somite stage (44 h)*. At 24-somite stage, the lens primordium can easily be seen. The heart begins to develop, and myotomal contractions start to occur (Figure 5G, K).

*Pre-hatching stage (50 h)*. The eye and lens have expanded in size and the head thickens due to brain growth. At this point, the three brain vesicles - the forebrain, midbrain, and hindbrain - have become structurally differentiated and can easily be distinguished (Figure 5L). Muscle contractions become more frequent. Embryos hatch between 50 and 60 h. Because hatching is variable, it is not particularly useful as a staging index. The tail is still curled and the head is bent around the yolk. The elongated, tube-shaped heart, which cannot yet be morphologically divided into atrium and ventricle, starts to beat at this stage (Figure 5H, L).

### Post-Hatching period (66-168 h)

**Figure 6:**
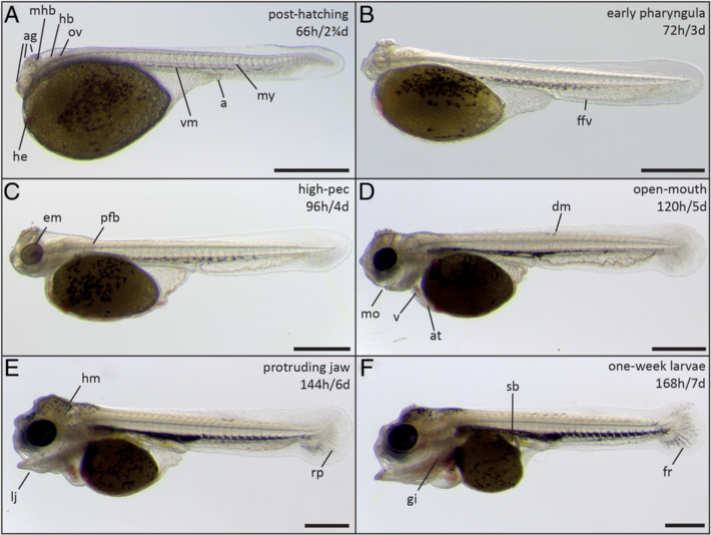
**Larvae in early post-hatching stages (66-168 h). (A)** post-hatching stage (66 h); **(B)** early pharyngula period (72 h); **(C)** high-pec stage (96 h); **(D)** open-mouth stage (120 h); **(E)** protruding-jaw stage (144 h); **(F)** one-week larvae (168 h). Abbreviations: a, anus; ag, adhesive glands; at, atrium; dm, dorsal melanophore (stripe); em, eye melanophores; ffv, fin fold veins; fr, fin rays; gi, gills; he, heart; hb, hindbrain; hm, head melanophores; lj, lower jaw; mhb, midbrain-hindbrain boundary; mo, mouth opening; my, myomeres; ov, otic vesicle; pfb, pectoral fin bud; rp, rays primordia; sb, swim bladder; v, ventricle; vm, ventral melanophore (stripe). Scale bars = 1 mm.

**Figure 7:**
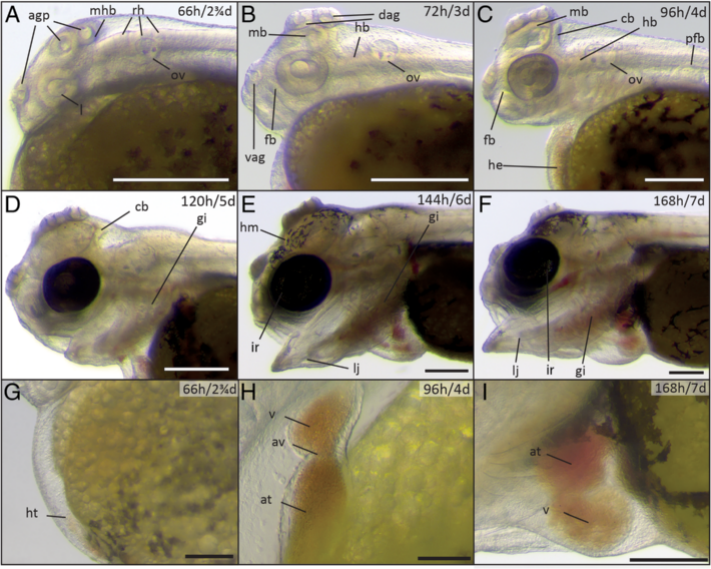
**Head and heart development in post-hatching stages (66-168 h). (A)** post-hatching stage (66 h); **(B)** early pharyngula period (72 h); **(C)** high-pec stage (96 h); **(D)** protruding-jaw stage (120 h); **(E)** open-mouth stage (144 h); **(F)** one-week larvae (168 h). **(G-I)** The developing heart at 66 h **(G)**, 96 h **(H)** and 168 h **(I)**. Abbreviations: agp, adhesive gland primordium; at, atrium; av, atrio-ventricular valve; cb, cerebellum; dag, dorsal adhesive gland; fb, forebrain; gi, gills; he, heart; ht, heart tube; ir, iridophores; l, lens; lj, lower jaw; hm, head melanophores; mb, midbrain; mhb, midbrain-hindbrain boundary; ov, otic vesicle; pfb, pectoral fin bud; rh, rhombomeres; v, ventricle; vag, ventral adhesive gland. A-F, I: Scale bar = 500 μm; G: Scale bar = 200 μm; H: Scale bar = 100 μm.

**Figure 8:**
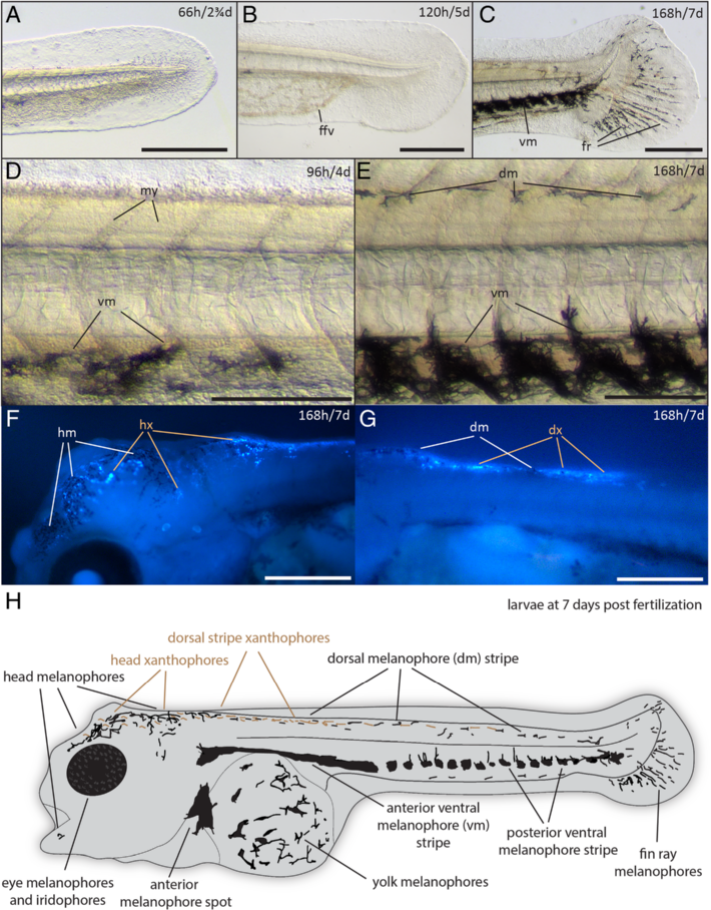
**Detail of tail, melanophore and xanthophore development in post-hatching stages (68-168 h). (A-C)** Caudal fin development at post-hatching stage (66 h, A), protruding-mouth stage (120 h, B) and one-week larvae stage (168 h, C). **(D, E)** Formation and dendrite extension of melanophores at high-pec stage (96 h, D) and one-week larvae stage (168 h, E). **(F, G)** Xanthophores on head **(F)** and in the dorsal stripe above the yolk **(G)** visualized under UV light. (**H**) Scheme summarizing the chromatophore distribution at 168 h/7d. Abbreviations: dm, dorsal melanophore (stripe); dx, dorsal xanthophores; hm, head melanophores; hx, head xanthophores; ffv, fin fold veins; fr, fin rays; my, myomeres; vm, ventral melanophore (stripe). A-C, F, G. Scale bars: 500 μm. D-E. Scale bars: 250 μm.

In the four days after hatching, the embryos (now referred to as larvae, or fry) rapidly start to develop the paired fins and craniofacial skeleton (Figures 6, 7, and 8). The rudiments of all organs are present and their morphogenesis (organogenesis) continues until the end of the first week of development.

*Post-hatching stage (66 h)*. Melanophores start to form ventrally in what is called the “ventral stripe” in zebrafish [43]. Unlike in zebrafish, no melanophores are formed in the dorsal and lateral stripe during the early stages of development (Figures 6A, 7A, 8A). In contrast to zebrafish, but similarly to medaka, stickleback and other cichlids [24,26,29,44], the yolk has no posterior extension. In zebrafish, a small projection of the yolk extends posteriorly up towards the anal region [22]. Still, the yolk sac is not completely round, and forms a cone-like tip at the posterior end. Further posterior to the end of the yolk sac, the digestive tract and anus can be seen. Shortly after hatching, the adhesive gland apparatus forms. The apparatus consists of two pairs of glands in the dorsal head region above the midbrain (dorsal glands) and one pair of glands anterior to the eye (ventral gland) (Figure 7A-F). They are used by the larvae to attach to the substrate and to each other before they reach the free-swimming stage, as previously described in cichlids [29,45,46] and the cave fish *Astyanax mexicanus* [46]. Under the described laboratory conditions in petri dishes, larvae mainly stick to particles such as remnants of the chorion that remain in the dish after hatching. Thereby, groups of larvae may all connect to a single particle and group together. The first red blood cells start to move through the circulatory system; this allows for the better visualization of the developing heart, which retains its tube-shaped form (Figure 7G).

*Early pharyngula period (72 h/3 d)*. The brain vesicles increase in size resulting in a further thickening of the head region (Figures 6B, 7B) and the isthmus, the connection between mid- and hindbrain, becomes more prominent (Figure 7B). The head starts to lift from the yolk. Also, vascularization starts along the ventro-caudal part of the medial fin fold (caudal aorta and caudal vein) (Figure 6B). The tail has straightened and the surface of the fin fold has increased, especially ventrally (Figure 6B). The adhesive glands are becoming more prominent at this stage (Figure 7B).

*High-pec stage (96 h/4 d)*. Melanophores start to form in the eye (Figures 6C, 7C), but only a few can be seen along the ventral zone of the body axis (Figure 8D). The pectoral fins can be seen as elongated blade-shaped tissues projecting dorsally from the yolk. Head and body axes now have nearly the same orientation and the head lifts up from the yolk (Figure 6C). The brain ventricles, midbrain and isthmus can be seen more clearly (Figure 7C). Also, the heart can now be morphologically separated into the ventricular and atrial chamber, separated by the atrio-ventricular valve. The cardiac looping is already in progress, moving the atrium to a more dorsal position and transiently generating an S-shaped structure, comparable to heart development in zebrafish [47,48] (Figure 7H).

*Open-mouth stage (120 h/5 d)*. A few melanophores start to form dorsally, and eye melanophores have increased in density, causing the eye to become opaque (Figure 6D). Silvery reflective iridophores can be detected in the eye and become more prominent after the fifth day of development (Figure 7E, F). The mouth opening and gills become visible (Figure 7D). Also, the chambers of the heart have become fully differentiated (Figure 6D) and the caudal fin starts to form, acquiring a more rounded shape (Figure 8B).

*Protruding-jaw stage (144 h/6 d)*. More melanophores form dorsally and appear for the first time in the head region. Also the first few xanthophores start to appear after day six, and can be detected under UV light (see Methods). They form both dorsally to the yolk and in the head region. They become more prominent after seven days of development (Figure 8F-G). The ventral melanophores condense in the posterior part of the body, giving them a segmented appearance that correlates with myomere position while anteriorly and also dorsally to the heart they are a coherent mass of cells (Figure 6E). The lower jaw extends anteriorly, stretching the head in a more anterior direction (Figure 7E). The caudal fin starts to develop fin rays that are readily populated by melanophores (Figure 6E). Compared to earlier stages, the strong vascularization in the ventral medial fin fold becomes less evident (Figure 6E).

*One-week larvae (168 h/7 d)*. The larva further increases in size, and the gills can be seen more clearly than in previous stages. The jaw becomes thicker and more strongly vascularized, and the larva is able to open and close its mouth freely (Figures 6F, 7F). The melanophores increase in number, and they aggregate more clearly (Figure 8C, E, H). Some of them project dendrites dorsally into the space between two myomeres. Xanthophores can now be detected both on the head and in the dorsal stripe in close proximity to melanophores (Figure 8F, G). They appear colorless until day seven both in reflected and under transmitted light, and can only be detected using UV-light (see Methods). Silvery reflective iridophores are less prominent than in zebrafish, medaka and tilapia [22,23,26] and can only be detected in the eye (Figure 7E, F; Figure 8H). The caudal fin rays have become thicker, and elongated melanophores are arranged around them (Figure 8C). The heart is now fully developed and can be divided into the sinus venosus, atrium, ventricle, and bulbus arteriosus [47,48]. The ventricular walls have thickened, indicated by the reduced visibility of red blood cells (Figure 7I). The swim bladder develops on the ventral side of the body, dorsal to the posterior end of the yolk plug (Figure 6F). Between days seven and eight, the swim bladder inflates and the larva begins to swim upright.

### Midas cichlid development is greatly influenced by temperature

**Figure 9:**
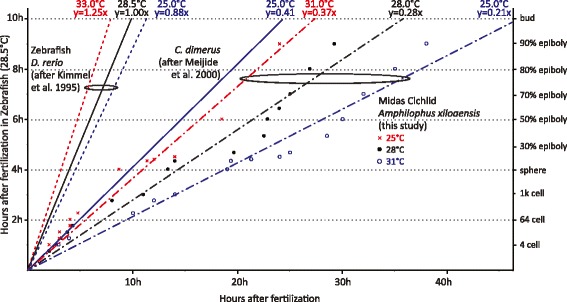
**The effects of temperature on developmental time.** Developmental rates in *D. rerio* (zebrafish) at 25, 28.5 and 33°C (from [22]), *C. dimerus* (South-American substrate-brooding cichlid) at 25°C (from [29]) and the Midas cichlids at 25, 28 and 31°C (this study), standardized to zebrafish development at 28.5°C (from [22]).

**Figure 10:**
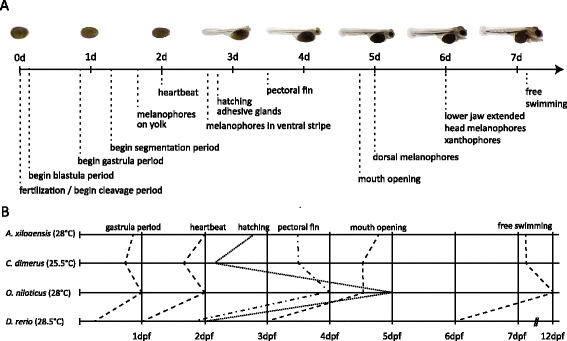
**Summary of Midas cichlid development and comparison to the development of other teleosts. (A)** Summary of the most important steps of the first week of Midas cichlid development at 28°C. **(B)** Comparison between this study and three further studies on teleosts including *C. dimerus*, a South American cichlid [29], *O. niloticus*, an African cichlid [26] and *D. rerio*, the zebrafish [22].

The early development of the Midas cichlid is slower than that of teleost genetic models such as medaka and zebrafish. However, it is comparable to the African Nile tilapia (Figure 9). We compared the homologous developmental stages to the South American cichlid *Cichlasoma dimerus* and the zebrafish *Danio rerio.* We show that, when raised at the same temperature (25°C), the developmental rate between fertilization and 100% epiboly is approximately two times slower than in *C. dimerus* and over four times slower than in zebrafish (Figure 9, Figure 10B). In particular, the rate of epiboly seems to be decelerated compared to zebrafish, a phenomenon that might be related to the large egg size. The influence of temperature on developmental rate is far greater than in zebrafish, where there is only a 1.42-fold difference between embryos developing at 25°C and 31°C [22]. In Midas cichlids, the difference is 1.76-fold. Despite this, later developmental stages seem to be less affected by temperature, with only minor differences in hatching time, development of pectoral fins and mouth opening between *C. dimerus* and *A. xiloaensis* (Figure 10A, B).

## Discussion

We describe the embryonic and larval development of *Amphilophus xiloaensis* as a representative of the Midas cichlid species complex. Midas cichlids are an excellent example of rapid adaptation and fast speciation in sympatry [49,50] and parallel evolution [19,51]. They allow us to integrate studies of genomics, development, adaptive radiation and phenotypic divergence into the field of “evo-devo”. The rate of divergence in many traits, including coloration and pigmentation, morphology of body shapes, lips, jaws and teeth as well as neural systems such as vision is much higher in cichlid fishes than in most other vertebrate groups [2,19,52]. In Nicaraguan crater lakes, speciation and phenotypic diversification took place over a very short time and endemic species have been described even in crater lakes that are less than 2000 years old [53]. At least eleven species have evolved in less than 25,000 years, carrying various traits that are divergent from the ancestral population [5,18,39]. Studies from other cichlids [21,41,54-60] and from sticklebacks [61-63] suggest that a few mutations of major effect are expected to play important roles in driving phenotypic richness and ecological diversity. The mutation rate of Midas cichlids has been estimated to be between 6.6 × 10^−8^ and 7.1 × 10^−8^ mutations per nucleotide per generation, comparable to the vertebrate average [53,64]. Still, genetic differences between different Midas cichlid species are small due to their long generation times (a conservative estimate is one year [64]) and recent time of divergence (2,000-25,000 years [15]).

Differences in developmental pathways [65-67] are often involved in the basis of ecologically relevant phenotypic differences [66] such as those observed in the Midas species complex, including body shape and craniofacial shape [68-70], lip shape [51,71,72], coloration [72-75] and pharyngeal jaw morphology [2] (Figure 1C, Table 1). Although most of these phenotypes arise later during ontogeny, differences might be already detectable on a subtle morphological and gene expression level – especially for craniofacial phenotypes associated with benthic-limnetic differences, as recently shown in other species (e.g. craniofacial skeleton of Malawi cichlids [57] or the Arctic charrs [76]). To examine if inter-species morphological and gene expression differences are indeed already present at early stages of Midas cichlid development, a standardization of embryonic timing and a comprehensive - and comparative -staging system are necessary as a baseline for hypothesis-driven research in this field. This staging forms the basis for future comparative developmental work on different species of Midas cichlids and closely related Neotropical cichlids [16]. Since development is greatly influenced by temperature, easily recognizable landmarks, along with a standardized temperature-time protocol, must be defined. This will ease the collection of comparable stages for molecular biological experiments, such as *in situ* hybridization or RNA extraction.

In Midas cichlids, embryonic traits such as the prominent adhesive glands and the early melanophore and xanthophore patterns differ from other model teleosts such as medaka and zebrafish. Adhesive glands have recently been studied in the cavefish *Astyanax mexicanus* (divergence time approximately 265 million years [77]) and described in other cichlids [29,45,46], but are not present in medaka and zebrafish.

The embryonic melanophore patterns we observe are very different from model teleosts such as medaka and zebrafish [22,23,44]. The prominent dorsal and lateral melanophore stripes are almost completely absent in Midas cichlid embryos, suggesting different migration patterns of neural crest cells, which are thought to generate all but the yolk melanophore in teleosts [78]. It has been proposed that some melanophores migrate from the yolk to populate the embryo, especially in the ventral zone; however, there has been some controversy surrounding this claim [32]. Further histological analysis using neural crest markers could solve this controversy and clarify the genetic cause of the different melanophore patterns observed in Midas cichlids.

The embryos of substrate brooders such as the Midas cichlid are also easier to use for genetic manipulations such as transgenesis than the massive, yolky eggs of mouth-brooding cichlids from the African Great Lakes, although these were the first species in which transgenesis was successfully performed [79,80]. Functional assays like those performed in zebrafish can also be carried out in Midas cichlids (Kratochwil CF, Sefton MM, Meyer A, unpublished results). Large clutch sizes and the slow development before the one-cell stage allow for the injection of considerable amounts of eggs. This method will allow researchers to transiently map the influence of gene overexpression or the activity of regulatory elements. Additionally, genetic manipulations by morpholinos or CRISPR-Cas, both of which have been shown to work in the Nile tilapia [81,82], might also be applicable in the Midas cichlid. Transcriptomic and genomic data sets, including a high-quality draft genome of *A. citrinellus* and low-coverage genomic information of eleven Midas cichlid complex species [6], is available to support these functional explorations (Table 1). One limitation is the long generation time - about nine to twelve months under laboratory conditions. Despite this drawback, it may still be possible to generate stable transgenes or knockouts, which would be relevant for experiments in Neotropical, substrate-brooding cichlids. Furthermore, the Midas cichlid could also serve as an excellent outgroup for the African cichlid species flocks, allowing for functional screens or assays of genes and cis-regulatory elements [83,84]. As shown here, Midas cichlids can be easily maintained, bred, stripped and raised in large numbers under laboratory conditions.

## Conclusions

It is still not fully understood which genes and mutations underlie the parallel evolution of traits and the quickly-evolving species richness exhibited by Midas cichlids. This study adds valuable information about the course of early development to help tackle questions about the molecular basis of phenotypic novelties from an evolutionary-developmental, evo-devo angle. The staging system in a representative Midas cichlid species will serve as a foundation for future experiments and ease interspecies comparisons. It will help to reproducibly select standardized developmental stages during development to analyze gene function and differences in gene expression and patterns. Midas cichlids have embryonic traits (adhesive glands, melanophore patterns) that differ from the classical developmental model teleosts, medaka and zebrafish. It will be interesting to analyze the genetic causes of these differences. We also propose the Midas cichlid as a new model organism for evolutionary developmental research. In addition to the availability of genetic resources and the possibility to perform functional experiments, Midas cichlids and their adaptively relevant phenotypic diversity are well-described from an ecological and evolutionary standpoint. These advantages, taken together, make this system very attractive for evolutionary-developmental questions.

## Methods

### Maintenance of adult fish

Adult Midas cichlids of *Amphilophus xiloaensis* (wild caught from crater Lake Xiloá, Nicaragua in 2010) were kept under constant conditions (28 ± 1°C, 12 h dark/light cycle, pH 8.5 ± 0.5) in 480 L (113.5 (length) × 50 (height) × 85 cm (depth)) or 550 L (110 × 50 × 100 cm) tanks. Two to five pairs are usually kept per tank to minimize aggressive behavior while maximizing reproductive success. Gravel was used as a substrate for the tanks. Each tank was equipped with clay flower pots split into halves as spawning substrate (Figure 1B). Cichlids are able to use the pots to hide, reducing stress and the frequency of attacks between fish. Pairs usually occupy one of the pots as their territory. If eggs are not removed by stripping, the female deposits her eggs on the inside of the pots, where the male fertilizes them. Specimens analyzed in this study were obtained both by regular spawning (eggs can be easily removed from pots) and stripping combined with *in vitro* fertilization.

### Stripping of eggs and fertilization

To obtain eggs, it is crucial that the fish are stripped at the right time. As soon as couples pair up and begin to defend their territory, the female must be checked daily for further behavioral and physiological changes. A few days before spawning, both the male and female become more aggressive. The female’s genital papilla swells, protrudes and turns reddish in color. Females showing these signs were removed from the water with a net and the eggs were stripped by applying light pressure to the abdominal region anterior of the genital papilla, followed by a slight squeezing movement towards the genital pore. Eggs should come out easily; if not, the female is not yet ready to spawn. If only a few eggs come out, it is likely that the eggs are not yet mature. Eggs were stripped directly into a petri dish (diameter 90 mm) filled with tank water. Between 400 and 1100 eggs can be obtained using this method (averaging around 700). Females spawn regularly (every four to six weeks) throughout the year.

Since there are no clear external signs to indicate the maturity of the males, we usually obtained sperm from one to three males. Stripping was performed using the same method as for females. We found no way to confirm that sperm was obtained, but in most cases (five out of six clutches collected) eggs were fertilized; the combination of survival rate and fertilization rate was estimated to be between 30 and 90% at three days post fertilization (d) at 28°C. The experiments were performed in accordance with the rules of the animal research facility of the University of Konstanz, Germany and have been granted permission by the animal care committee (Regierungspräsidium) Freiburg, Germany (Az. 35–9185.81/G13/99).

### Raising conditions

After fertilization, eggs were kept for five minutes in the petri dish, which is sufficient for successful fertilization. Next, eggs were transferred into a new dish containing clean, autoclaved tank water. The eggs were distributed into multiple petri dishes (50 eggs per plate) and kept in a 28°C incubator (HIR10M Grant, Boekel) or in 25°C or 31°C water baths (1003, GFL), without agitation or aeration. The embryos were moved into fresh petri dishes with new autoclaved tank water every 24 hours.

### Visualization of xanthophores

To visualize xanthophores in developing Midas cichlids, we used a modified version of the method described for zebrafish and African cichlids [85,86]. Embryos were mounted in 3% methylcellulose in autoclaved tank water (1000 μl) mixed with ammonium hydroxide solution (20 μl) and β-mercaptoethanol (1 μl). We verified that the pH was above pH9 using pH indicator strips (Macherey-Nagel). Auto-fluorescence could only be detected with the addition of ammonium hydroxide and β-mercaptoethanol under UV light. Without the solutions (i.e. in methylcellulose alone), no auto-fluorescence could be detected. Furthermore, the cells we identified as xanthophores did not show auto-fluorescence under blue light as e.g. shown for leucophores that are similar to xanthophores in their developmental specification and differentiation [87].

### Image acquisition

Photographs were taken with a stereomicroscope (Leica MZ10 F with Leica DMC2900 Camera) using the Leica Application Suite software 4.5.0. To improve the depth of field, we used the “Multifocus Montage” module/plugin of the Leica Application Suite software. Six to eight photographs at different focal positions were matched and combined, retaining the best-focused parts of each photograph and resulting in a single sharp image. Images of UV epiluminescence were taken with a Zeiss AxioCam Mrc digital camera using a Zeiss SteREO Lumar V.12 Stereomicroscope with UV filter. Photographs of adult fish were taken with a Canon EOS 7D SLR with a 17-40 mm lens.

## Abbreviations

a: Anus
ag: Adhesive glands
agp: Adhesive gland primordium
ap: Animal pole
at: Atrium
av: Atrio-ventricular valve
b: Blastodisc
bd: Blastoderm
bm: Blastomeres
br: Brain
cb: Cerebellum
ch: Chorion
cp(s): Cleavage plane(s)
d: Days post fertilization
dag: Dorsal adhesive gland
dm: Dorsal melanophore (stripe)
dx: Dorsal xanthophores
ea: Embryonic axis
em: Eye melanophores
es: Embryonic shield
ez: Evacuation zone
fb: Forebrain
ffv: Fin fold veins
fr: Fin rays
ft: Filament tuft
gi: Gills
gr: Germ ring
h: Hours post fertilization
hb: Hindbrain
he: Heart
hm: Head melanophores
ht: Heart tube
hx: Head xanthophores
ir: Iridophores
l: Lens
lj: Lower jaw
mb: Midbrain
mhb: Midbrain-hindbrain boundary
mi: Micropyle
ml: Mucous layer
mo: Mouth opening
my: Myomeres
opr: Optic primordium
ov: Otic vesicle
pfb: Pectoral fin bud
ps: Perivitelline space
rh: Rhombomeres
rp: Rays primordia
s: Somites
sb: Swim bladder
tb: Tailbud
v: Ventricle
vag: Ventral adhesive gland
vm: Ventral melanophore (stripe)
vp: Vegetal pole
y: Yolk
ym: Yolk melanophores
yp: Yolk plug
